# Trajectory Correction and Locomotion Analysis of a Hexapod Walking Robot with Semi-Round Rigid Feet

**DOI:** 10.3390/s16091392

**Published:** 2016-08-31

**Authors:** Yaguang Zhu, Bo Jin, Yongsheng Wu, Tong Guo, Xiangmo Zhao

**Affiliations:** 1 Key Laboratory of Road Construction Technology and Equipment of MOE, Chang’an University, Xi’an 710064, China; wuyongsheng@chd.edu.cn (Y.W.); guotong@chd.edu.cn (T.G.); 2 State Key Laboratory of Fluid Power & Mechatronic Systems, Zhejiang University, Hangzhou 310028, China; bjin@zju.edu.cn; 3 School of Information Engineering, Chang’an University, Xi’an 710064, China; xmzhao@chd.edu.cn

**Keywords:** hexapod, semi-round rigid foot, kinematics, trajectory correction, sensors system

## Abstract

Aimed at solving the misplaced body trajectory problem caused by the rolling of semi-round rigid feet when a robot is walking, a legged kinematic trajectory correction methodology based on the Least Squares Support Vector Machine (LS-SVM) is proposed. The concept of ideal foothold is put forward for the three-dimensional kinematic model modification of a robot leg, and the deviation value between the ideal foothold and real foothold is analyzed. The forward/inverse kinematic solutions between the ideal foothold and joint angular vectors are formulated and the problem of direct/inverse kinematic nonlinear mapping is solved by using the LS-SVM. Compared with the previous approximation method, this correction methodology has better accuracy and faster calculation speed with regards to inverse kinematics solutions. Experiments on a leg platform and a hexapod walking robot are conducted with multi-sensors for the analysis of foot tip trajectory, base joint vibration, contact force impact, direction deviation, and power consumption, respectively. The comparative analysis shows that the trajectory correction methodology can effectively correct the joint trajectory, thus eliminating the contact force influence of semi-round rigid feet, significantly improving the locomotion of the walking robot and reducing the total power consumption of the system.

## 1. Introduction

Multi-legged walking machines, compared to those with wheeled or tracked locomotion, are widely recognized as a much more effective and efficient form of transportation vehicle, especially on complex and unstructured terrains. Hexapod robots, one such type of legged walking machines, generally have superior performance than those with fewer legs in terms of less complexity of the control method, more statically stable walking, and faster walking speed [1,2]. Therefore, in recent years, multi-legged walking robots have been extensively researched and are noted for their good environmental adaptability and movement flexibility. The potential application terrain for use of these robots is mainly unstructured surface environments [3].

In order to have sufficient adaptive capacity for different conditions, the design of the robotic leg and foot is becoming particularly important. According to current research on multi-legged walking robots, foot designs, on the basis of the different linkages between the leg and foot tip, can be divided into two main groups; non-articulated feet and passive ankle feet. The non-articulated foot group can be farther divided into flat foot, rounded flat foot, (semi)-round foot, and so on. The robots Dante II [4], AMBLER [5], and ROWER [6] have been built with flat foot designs, while the G. E walking Truck [7] and Airbug [8] robots adopted rounded flat foot designs. Other robots such as MASCHA [9], Silex [10], LauronIII [11], TUM [12], TARRY II [13], and so on use (semi)-round rigid feet. However, the robots with a passive ankle foot design have a large difference in the number of degrees of freedom (DOF). PV II [14], TITAN [15], and OSU [16] adopted a passive ankle-joint with a single DOF, while the COMET II [17] and SILO4 [18] adopted a foot structure with an ankle-joint, which has two revolute joints. The flat feet of HAMLET [19] are connected with the body by a spherical hinge with three DOF. Overall, the foot with a passive ankle-joint is complex in its design and manufacturing, and the cost is also correspondingly higher. In addition, the trajectory of robots with movable ankle-joints and flat feet is also limited by foothold. The small angle between the foot trajectory and the surface will likely cause impact with the ground, or even stumbling. The non-articulated foot with a flat or round structure also has great limitations in its adaptability to complex terrain. By contrast, a (semi)-round rigid foot has significant advantages in applications, so it is widely used in many robot prototypes [20,21,22].

When designing the size of a semi-round rigid foot, using a smaller radius can cause the foot to sink into a surface, and should be considered because the walking robot usually has to adapt to a variety of complex surface environments, such as rugged or soft ground. This problem can be effectively alleviated by increasing the radius of the foot, but on hard ground a large-radius foot will change the contact point between the foot and the ground when it is in the supporting phase, thus affecting the trajectory. Because multi-legged robots have a parallel closed chain structure in the standing state, the nonconformity of body trajectory error caused by the semi-round foot of each leg will eventually lead to sliding between the foot and the ground, and affect the stability of the robot. To address the problem above, Chen et al. proposed a trajectory correction method [23], where the predefined trajectory of the robotic foot tip is modified according to the information from force feedback. In this way, the robot can adapt to the current terrain at any time and it can relieve the running deviation. In another paper [24], when the running deviation rate of the robot reaches a gate value, the robot can amend it by adjusting gait or changing direction. Wei et al. [25] proposed a method to measure the deviation degree of the body during movement by using machine vision. More specifically, they used characteristic points of machine vision to track external parameters of the camera during movement between two frames obtained by pose estimation technology. On this basis, calculation of the deviation degree was completed. Kwon et al. [26] proposed an adaptive trajectory generation method for quadruped robots with semicircular feet to control body speed and heading. The adaptive gait patterns are changed by the sequential modulation of the locomotion period and the stride per step, which are determined by the desired body speed and heading commands. The researchers above have achieved the correction from the closed-loop control of the trajectory, but the problem of deviation between the actual position and the desired trajectory of the robot's feet have not been solved. Guardabrazo et al. [27] had proposed a method to solve it. However the kinematic model they established was mainly used to analyze the motion of the robot with mechanical legs imitating insects in a two-dimensional working plane. Although the problem of a semi-round rigid foot in a three-dimensional space has been investigated, a complete kinematic relationship is missing.

Due to the special structure of the semi-round foot, the deviation of each leg is different and it will further lead to interference between the legs, as well as more energy consumption. Therefore, it is necessary to propose a correction algorithm for the kinematics. In order to find a better way to solve the problem of deviation caused by a semi-round foot and to improve the accuracy of the inverse kinematic solution, the trajectory deviation of the body and leg caused by a semi-round foot is studied. According to the kinematic analysis, a correction methodology for forward/inverse kinematic solutions in three-dimensional space between the relative position of the semi-round foot and joint trajectory is proposed. A nonlinear mapping relationship between foot position and joint angle is approached by constructing the optimal linear regression function, and then the inverse kinematic relationship of a robot with semi-round foot is realized based on LS-SVM. Using the existing hexapod walking robot platform with multi-sensors, a series of related experiments are carried out, and the validity of the correction methodology for improving foot stress, body trajectory, foot slip, and energy consumption is verified.

The remainder of this paper is organized as follows: Section 2 provides a brief introduction to the robot system. Section 3 presents the modeling and the methods in detail. Section 4 describes the experiments and discusses the results. Section 5 summarizes and concludes the paper.

## 2. Description of the Structure and Sensor System

**Figure 1:**
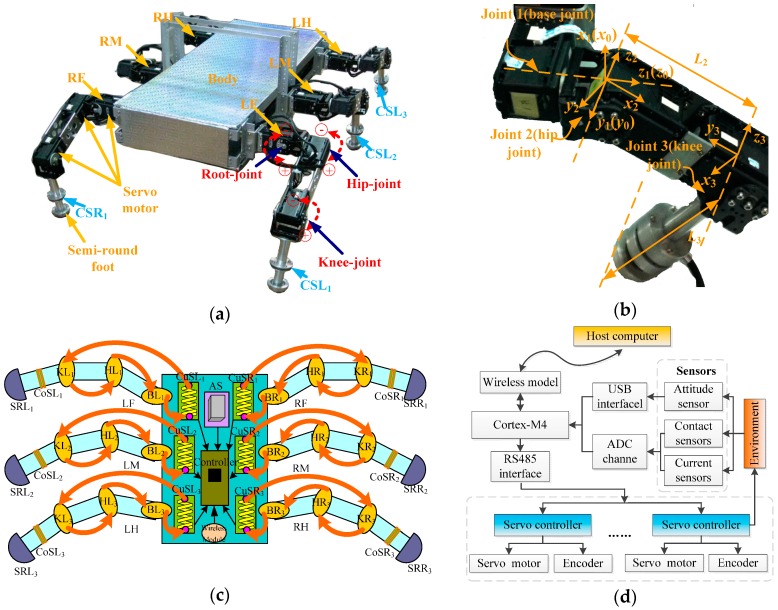
Structure and sensor system of the robot: (**a**) Mechanical model of the hexapod walking robot; (**b**) Reference coordinate system and corresponding joint variable of the three joint mechanical leg; (**c**) Distribution of the sensors; (**d**) Diagram of the control architecture.

The structure of a robotic leg should not only imitate the structure of legged animals (such as insects and spiders), but should also consider its power system and constraints [28]. In this paper, a type of leg structure with a mammalian configuration is adopted, since mammal legs require less joint torques to support the body [29]. Mammalian legs usually have three joints (base-joint, hip-joint, and knee-joint). The base-joint and the hip-joint are located higher than knee-joint. Its characteristics of low energy consumption and large loading are more suitable for outdoor tasks. The mechanical model of the robot is shown in Figure 1a. Each leg consists of three linkages which are connected by a hip joint and a knee joint. The leg mechanism is attached to the body via a base joint. Here, a semi-round foot with a certain radius is used. The leg variables are shown in Figure 1b. The robot can achieve translation and rotation in three-dimensional space in its workspace [30].

The sensors, especially the camera, attitude sensor, and force sensor, play an important role in the intelligent robotics field [31,32]. The distribution of the multi-sensor system of the robot for observing motion state is shown in Figure 1c. BLi, HLi, and KLi (BRi, HRi, and KRi for the *i*th leg) respectively represent the base-joint, hip-joint, and knee-joint on each left (right) leg, and LF, LM, and LH (RF, RM, and RH) respectively represent the front leg, middle leg, and hind leg on the left (right) side. Each joint is composed of a DC motor, a high reduction rate gear system, and has an integrated encoder, which is used for detecting the position of the joint angle. Contact sensors (CoSLi or CoSRi) installed on the semi-round foot tips (SRLi or SRRi) are used for monitoring the gait of the robot. In order to reflect the energy consumption of each leg in different gaits, current detection modules (CuSLi or CuSRi) are used. The arrows represent current flow direction. The attitude sensor (AS) is installed in the body to monitor the posture of the robot body. And then, the data processed by the wireless module transmits to a host computer to generate commands. Finally, the commands are send to the slave board Cortex-M4 to control the motion of each leg and to monitor the state of each sensor. The diagram of the control architecture is shown in Figure 1d.

## 3. Trajectory Correction Methodology

The deviation caused by semi-round rigid foot not only occurs in the vertical direction, but also the horizontal direction. For multi-legged parallel mechanisms, these inaccurate displacements will cause deviation between the real posture and ideal posture of the body, causing the walking robot to not move smoothly. In addition, each supporting leg of the robot will lead to different deviations when they are actuated at the same time. This will not only lead to interference of supporting legs for its redundant DOFs, but also waste a lot of system energy. When this situation persists, slipping will occur and will influence the stability of the robot [33]. Therefore, it is extremely necessary to put forward kinematic correction methodology according to the special structure of the semi-round rigid foot. 

### 3.1. Kinematic Analysis

Each leg can be regarded as a manipulator with three rotating joints attached to a stationary base. It includes two parallel joints, the hip-joint *θ*_2_ and the knee-joint *θ*_3_, which is connected to the body through a base-joint *θ*_1_. Therefore, the establishment of the kinematic model and the derivation of the kinematic equation can follow traditional robot technology and methods. The kinematic model of this paper is obtained by defining the reference coordinate system using the Denavit-Hartenberg method [34]. The model of the leg structure with three joints is shown in Figure 1b, in which the reference coordinate and the corresponding joint variables are marked. The base coordinate system $\sum_{0}^{\left(i\right)}(O_{0}^{\left(i\right)}\;-\;xyz)$ is located on the static robot body. The connection parameters of the D-H model are listed in Table 1.

Therefore, under the base reference coordinate system $\sum_{0}(O_{0}\;-\;xyz)$ of the leg, three joint angles are already known and foot position vector P^tip0 can be obtained by the forward kinematic equation, as follows:

(1) P^tip0 = [P^tip0xP^tip0yP^tip0z] = [C1C23L3 + C1C2L2S1C23L3 + S1C2L2S23L3 + S2L2],

in which, $S_{i}=\sin\theta_{i}$, $C_{i}=\cos\theta_{i}$, $S_{\text{ij}}=\sin(\theta_{i}+\theta_{j})$, $C_{\text{ij}}=\cos(\theta_{i}+\theta_{j})$, $\theta_{i}$ and $\theta_{j}$ are the mean joint angles of *i*th and *j*th, respectively. 

Here, the algebraic method is used to solve the inverse kinematic problem. The plus symbol is used for the front-leg of the multi-legged robot, and the minus symbol is used for the other leg:

(2) {θ1 = arctan(P^ytip0P^xtip0)θ2 = arcsinP^x2tip0 + P^y2tip0 + P^z2tip0 + L22 − L322L2P^x2tip0 + P^y2tip0 + P^z2tip0 + arctanP^y2tip0 + P^x2tip0P^ztip0θ3 = ± arccosP^x2tip0 + P^y2tip0 + P^z2tip0 − L22 − L322L2L3..

### 3.2. Kinematics Correction of a Single Leg with a Semi-Round Rigid Foot

**Figure 2:**
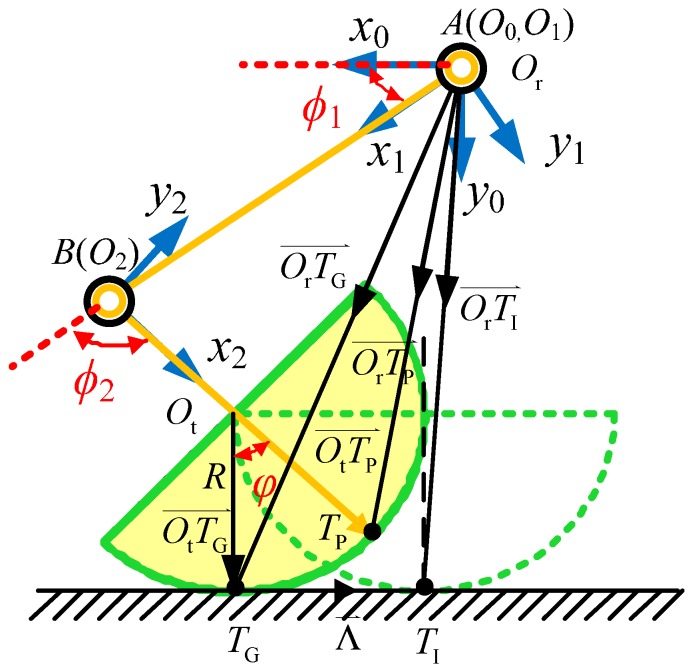
Side view of a single leg with a semi-round rigid foot.

The real trajectory of the body coincides with the theoretical trajectory when the foot is regarded as a point and there is no slipping. In theory, it can follow the ideal trajectory very well, but it is not practical for the mechanical structure. However, when the foot structure is semi-round, even though there is no slipping, the contact point between the foot and ground will be changing during the movement, which will cause deviation from the preset trajectory. Hence, in order to eliminate this deviation, the joint angle needs to be corrected. Because the robot is a closed kinematic chain, these deviations will lead to the error between the actual posture and the ideal posture of the robot body. Figure 2 is a side view of a single leg with a semi-round rigid foot. For ease of analysis, the concept of ideal foothold is proposed. On the horizontal surface, when the axial direction of the 3rd linkage of leg is perpendicular to the surface of the ground (shown in Figure 2 as a dashed line), the contact point of the horizontal surface and semi-round rigid foot on the ground is the ideal foothold *T*_I_, and this point on the semi-round rigid foot is called foot reference point *T*_P_. For the real foothold of the support leg at any posture, the only ideal foothold can be calculated by neglecting any small slippage between the foot and the ground, supposed as pure rolling. It is assumed that the origin of the base-joint coordinate is *O*_r_, the spherical center of the semi-round foot is *O*_t_, and the real contact point of the semi-round rigid foot and the ground is *T*_G_, which is shown in Figure 2. The angle between link 3 and the perpendicular of the horizontal plane is φ. The location of the ideal foothold in the base-joint coordinate system can be calculated by forward kinematics, as long as each joint angle vector is known.

According to the assumption that there is no relative slipping between the semi-round rigid foot and the ground, then:

(3) $$\left|\vec{\Lambda }\right|\;=\;\left|\vec{T_{G}T_{I}}\right|\;=\;\left|\overset{⌢}{T_{G}T_{P}}\right|,$$

in which, $\left|\overset{⌢}{T_{G}T_{P}}\right|$ is a circle between the foot reference point *T*_P_ and the real contact point *T*_G_. After vector analysis of Figure 2, the foot reference position is obtained:

(4) $$\vec{O_{r}O_{t}}\;=\;\left[\begin{array}{c} C_{1}C_{23}(L_{3}\;-\;R)\;+\;C_{1}C_{2}L_{2}\\ S_{1}C_{23}(L_{3}\;-\;R)\;+\;S_{1}C_{2}L_{2}\\ S_{23}(L_{3}\;-\;R)\;+\;S_{2}L_{2} \end{array}\right],$$

and real foothold is:

(5) $$\vec{O_{r}T_{G}}\;=\;\left[\begin{array}{c} C_{1}C_{23}(L_{3}\;-\;R)\;+\;C_{1}C_{2}L_{2}-R\\ S_{1}C_{23}(L_{3}\;-\;R)\;+\;S_{1}C_{2}L_{2}\\ S_{23}(L_{3}\;-\;R)\;+\;S_{2}L_{2} \end{array}\right],$$

As for ideal foothold, we have:

(6) $$\vec{O_{r}T_{I}}=\vec{O_{r}T_{G}}+\vec{\Lambda },$$

in which:

(7) $$\vec{O_{r}T_{I}}=\left[\begin{array}{c} C_{1}C_{23}(L_{3}-R)+C_{1}C_{2}L_{2}-R\\ S_{1}C_{23}(L_{3}-R)+S_{1}C_{2}L_{2}+\Lambda_{y}\\ S_{23}(L_{3}-R)+S_{2}L_{2}+\Lambda_{z} \end{array}\right],$$

because of:

(8) $$\vec{O_{t}T_{P}}\;=\;{\left[\begin{array}{ccc} C_{1}C_{23}R & S_{1}C_{23}R & S_{23}R \end{array}\right]}^{T},$$

(9) $$\vec{O_{t}T_{G}}\;=\;{\left[\begin{array}{ccc} -R & 0 & 0 \end{array}\right]}^{T}.$$

Therefore, the angle φ between the 3rd linkage and the perpendicular of the horizontal plane can be obtained by Equations (8) and (9):

(10) $$\cos\varphi \;=\;\frac{\vec{O_{t}T_{P}}\cdot \vec{O_{t}T_{G}}}{\vec{\left|O_{t}T_{P}\right|}\vec{\left|O_{t}T_{G}\right|}}\;=\;\frac{-C_{1}C_{23}R^{2}}{R^{2}}\;=\;-C_{1}C_{23},$$

(11) $$\Rightarrow \varphi \;=\;\arccos\left(-C_{1}C_{23}\right)$$

because:

(12) $$\left|\vec{\Lambda }\right|\;=\;R\cdot \varphi .$$

Usually, $\Lambda_{x}=0$, $\vec{\Lambda }$ and $\vec{O_{t}T_{P}}$ are coplanar, so:

(13) $$\vec{L}\;=\;{\left[\begin{array}{ccc} 0 & \Lambda_{y} & \Lambda_{z} \end{array}\right]}^{T}\;=\;{\left[\begin{array}{ccc} 0 & \frac{S_{1}C_{23}\varphi R}{\sqrt{S_{23}^{2}+S_{1}^{2}C_{23}^{2}}} & \frac{S_{23}\varphi R}{\sqrt{S_{23}^{2}+S_{1}^{2}C_{23}^{2}}} \end{array}\right]}^{T}.$$

Hence, in the base-joint coordinate system, the kinematics solution of the ideal foothold is obtained:

(14) OrTI⇀ = [T^Ix0T^Iy0T^Iz0] = [C1C23(L3 − R) + C1C2L2 − RS1C23(L3 − R) + S1C2L2 + ΛyS23(L3 − R) + S2L2 + Λz].

In the same way, when the position of the ideal foothold is known, each joint angle vector can be obtained by the inverse kinematics solution.

**Figure 3:**
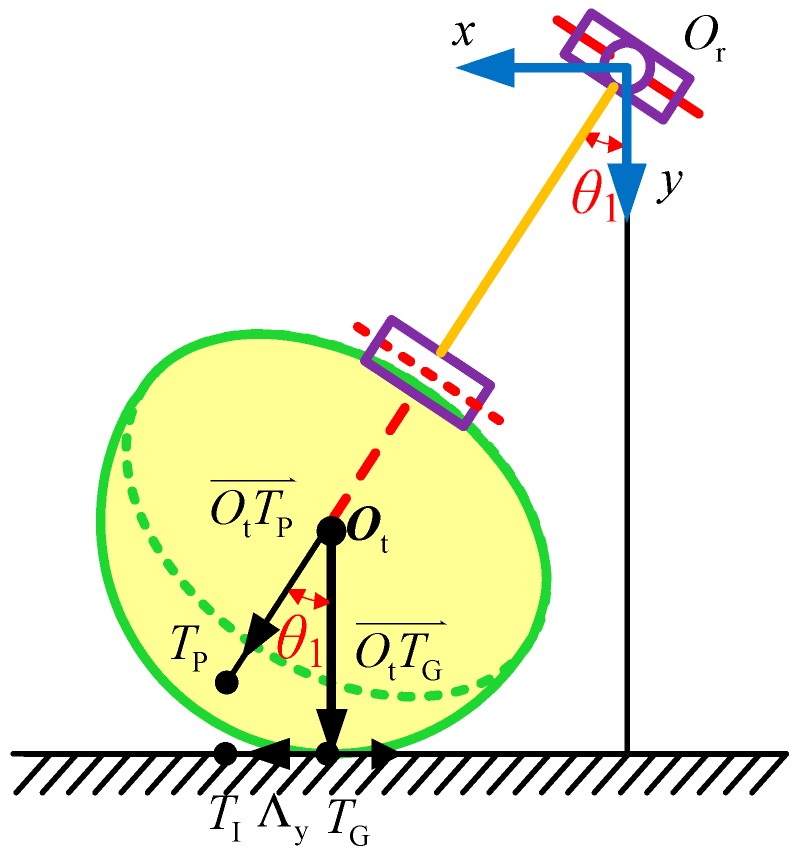
Front view of a single leg with a semi-round rigid foot.

The front view of a single leg with a semi-round rigid foot is shown in Figure 3, which shows that:

(15) T^Iy0 − ΛyT^Ix0 + R = tanθ1.

From Equation (15), we can obtain:

(16) θ1′ = arctanT^Iy0 − ΛyT^Ix0 + R.

Because of Equation (6), we have:

(17) [T^Ix0T^Iy0T^Iz0] = [P^xtip0 − C1C23R − RP^ytip0 − S1C23R + ΛyP^ztip0 − S23R + Λz]⇒P^tip0 = [T^Ix0 + C1C23R + RT^Iy0 + S1C23R − ΛyT^Iz0 + S23R − Λz].

Substituting Equation (17) into Equation (2), the modified joint angles $\theta_{2}^{\prime }$ and $\theta_{3}^{\prime }$ can be obtained. However, it is difficult to solve because Λ_x_, Λ_y_, and φ all have a relationship with $\theta_{2}^{\prime }$ and $\theta_{3}^{\prime }$. Here, it is approximately solved by using uncorrected joint angles. 

According to Equation (13), we have:

(18) $$\vec{\Lambda }\;\approx \;\left[\begin{array}{ccc} 0 & \frac{{\bar{S}}_{1}{\bar{C}}_{23}\varphi R}{\sqrt{{\bar{S}}_{23}^{2}+{\bar{S}}_{1}^{2}{\bar{C}}_{23}^{2}}} & \frac{{\bar{S}}_{23}\varphi R}{\sqrt{{\bar{S}}_{23}^{2}+{\bar{S}}_{1}^{2}{\bar{C}}_{23}^{2}}} \end{array}\right]{}^{T},$$

(19) θ2′ = arcsin(T^Iz0 − Λz + S¯23R)2 + (T^Iy0 − Λy + S¯1C¯23R)2 + (T^Ix0 + R + C¯1C¯23R)2 + L22 − L322L2(T^Iz0 − Λz + S¯23R)2 + (T^Iy0 − Λy + S¯1C¯23R)2 + (T^Ix0 + R + C¯1C¯23R)2−arctan(T^Iy0 − Λy + S¯1C¯23R)2 + (T^Ix0 + R + C¯1C¯23R)2T^Iz0 − Λz + S¯23R,

(20) θ3′=±arccos(T^Iz0−Λz+S¯23R)2+(T^Iy0−Λy+S¯1C¯23R)2+(T^Ix0+R+C¯1C¯23R)2−L22−L322L2L3,

where, ${\bar{S}}_{i}=\sin{\bar{\theta }}_{i}$, ${\bar{C}}_{i}=\cos{\bar{\theta }}_{i}$, ${\bar{S}}_{\text{ij}}=\sin\left({\bar{\theta }}_{i}+{\bar{\theta }}_{j}\right)$, and ${\bar{C}}_{\text{ij}}=\cos\left({\bar{\theta }}_{i}+{\bar{\theta }}_{j}\right)$, in which ${\bar{\theta }}_{i}\;\text{and}\;{\bar{\theta }}_{j}$ are the *i*-th and *j*-th joint-angles before correction. Upon substituting Equation (18) into Equations (19) and (20), the amended inverse kinematics relationship of a semi-round rigid foot is achieved. For a multi-legged robot, when it is used for the foreleg, Equation (19) has a positive sign, and when it is used for a rear leg, the equation has a negative sign.

### 3.3. Semi-Round Rigid Foot Trajectory Correction Algorithm Based on LS-SVM

The correction algorithm for the foot trajectory can eliminate the effect of semi-round rigid feet on the robot. However, the previous inverse kinematic algorithm is obtained by the approximation algorithm according to Equations (16)–(20), which has fast computational speed but the accuracy is not high, so the trajectory correction algorithm for a semi-round rigid foot based on the least squares support vector machine (LS-SVM) is proposed to solve this problem. The basic idea of SVM is mapping the input vector to a high dimensional feature space by using nonlinear transformation, and constructing an optimal decision function in this space [35]. When constructing the optimal decision function, the structural risk minimization principle is used and the point multiplication in the high-dimensional feature space is replaced by using the kernel function of the original space.

Assuming a given training sample ${\left\{x_{k},y_{k}\right\}}_{k=1}^{N},x_{k}\in R^{n},y_{k}\in R:$

(21) y(x) = 〈w,φ(x)〉 + b,

where 〈.,.〉 represents the point multiplication, and $w\in R^{n_{h}}$ is the weight vector of the original weighted space. $\varphi \left(\cdot \right):R\to R^{n_{k}}$ is a nonlinear mapping of samples from the original space to the high dimensional feature space, and the linear regression function is constructed in this space.

LS-SVM [36] is an extension of standard SVM. The optimization index adopts squared terms and the in-equation constraints of standard SVM are replaced by equation constraints. That means the quadratic programming problems transform into problems of the linear equation set solution. This method simplifies the complexity of the calculation and accelerates the solving process. The optimization problem of LS-SVM can be described as:

(22) $$\underset{w,e}{\lim}\vartheta (w,e)\;=\;\frac{1}{2}{∥w∥}^{2}\;+\;\gamma \frac{1}{2}\sum_{k=1}^{N}e_{k}^{2}.$$

The constraint condition is:

(23) $$y_{k}(x)\;=\;\langle w,\varphi (x_{k})\rangle \;+\;b\;+\;e_{k},k\;=\;1,\ldots ,N,$$

where $e_{k}\in R$ is error variable, and γ≥0 is a regular constant. A smaller γ can avoid over-fitting caused by noise. 

Introducing the Lagrangian function:

(24) $$\Gamma (w,b,e;\alpha )\;=\;\vartheta (w,e)\;-\;\sum_{k=1}^{N}\alpha_{k}\left\{\langle w,\varphi (x_{k})\rangle \;+\;b\;+\;e_{k}\;-\;y_{k}\right\},$$

in which, $a_{k}\in R$ are Lagrange multipliers, so the constraint conditions become:

(25) $$\{\begin{array}{l} \frac{\partial \Gamma }{\partial w}\;=\;0\to w\;=\;\sum_{k=1}^{N}\alpha_{k}\varphi (x_{k})\\ \frac{\partial \Gamma }{\partial b}\;=\;0\to \sum_{k=1}^{N}\alpha_{k}\;=\;0\\ \frac{\partial \Gamma }{\partial e_{k}}\;=\;0\to \alpha_{k}\;=\;\gamma e_{k}\;,\;k\;=\;1,\ldots ,N\\ \frac{\partial \Gamma }{\partial \alpha_{k}}\;=\;0\to \langle w,\varphi (x_{k})\rangle \;+\;b\;+\;e_{k}\;-\;y_{k}(x),\;k\;=\;1,\ldots ,N \end{array}.$$

Those constraints are consistent with the optimal conditions of standard SVM except $a_{k}=\gamma e_{k}$. The linear equation can be obtained by eliminating the variables *w* and *e*_k_:

(26) $$\left[\begin{array}{cc} 0 & e^{T}\\ e & \Omega +\gamma^{-1}I \end{array}\right]\left[\begin{array}{c} b\\ \alpha \end{array}\right]\;=\;\left[\begin{array}{c} 0\\ y \end{array}\right],$$

where, I is a unit matrix with *n × n*, *y* = [*y*_1_,…,*y_N_*], *e* = [1,…,1], *α* = [*α*_1_,…,*α_N_*]. According to the Merce condition, we have:

(27) $$\Omega_{\text{kl}}\;=\;\langle \varphi (x_{k}),\varphi (x_{l})\rangle =\;\Psi (x_{k},x_{l}),\;k\;=\;1,\ldots ,N.$$

Although the selection criteria of the kernel function Ψ(*x*_k_*,x*_l_) is consistent with the standard SVM, the radial basis function is widely used now, so the linear regression function is:

(28) $$\Psi (x,x_{k})\;=\;\exp\left\{\;-\;\frac{{\left\|x\;-\;x_{k}\right\|}^{2}}{2\sigma^{2}}\right\},$$

in which the term σ is the kernel bandwidth.

(29) $$y_{k}(x)\;=\;\sum_{k=1}^{N}\alpha_{k}\Psi (x,x_{k})\;+\;b,$$

where *α* and b satisfy Equation (26).

The forward/inverse kinematic solution of the ideal foot is derived under the base-joint coordinate system in the last section. An approximate solution of inverse kinematics is obtained by mapping the idea location of the foot to the angle-joint. This process can be represented by:

(30) (P^xtip0,P^ytip0,P^ztip0)→(θ1,θ2,θ3).

In order to solve this nonlinear mapping problem, LS-SVM is utilized to approximate the mapping in this section. In other words, this nonlinear mapping between the ideal location of the foot (P^xtip0,P^ytip0,P^ztip0) and the joint angle ($\theta_{1},\theta_{2},\theta_{3})$ is approached by constructing the optimal linear regression function, and then the inverse kinematic solution is found.

**Figure 4:**
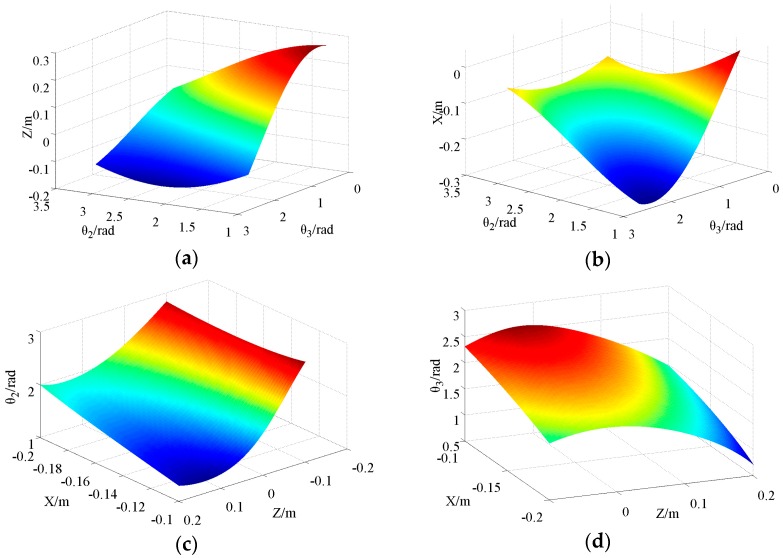
Input/output curved surface of the forward/inverse kinematic model according to the correction algorithm: (**a**) Forward kinematics Z; (**b**) Forward kinematics X; (**c**) Inverse kinematics *θ*_2_; (**d**) Inverse kinematics *θ*_3_.

**Figure 5:**
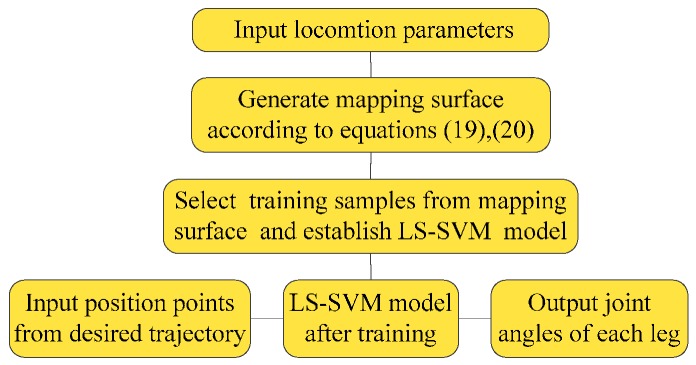
Process of the inverse kinematics solution by LS-SVM.

The forward kinematics model can be obtained directly by Equation (17). However, the result of inverse kinematics is an approximate solution, which can be applied in low precision occasions, but it may cause locomotion error when used in high precision occasions. Therefore, when choosing training samples, the results will be closer to the ideal solution after iteration computation by the approximate method. In this way, the training sample obtained is more accurate, and the accuracy of the training result is higher. From Equation (16) to Equation (20), Λ_y_, Λ_z_, and φ are all related to $\theta_{2}^{\prime }$ and $\theta_{3}^{\prime }$, which makes it difficult to obtain analytical solutions directly. In general, only an approximate solution is achieved according to the uncorrected joint rotation angle. This result is usually substituted into Equations (19) and (20), which are the kinematics inverse solutions of $\theta_{2}^{\prime }$ and $\theta_{3}^{\prime }$ after correction of the semi-round foot. Here, the values of $\theta_{2}^{\prime }$ and $\theta_{3}^{\prime }$ obtained by using the iterative method are substituted into Equations (19) and (20), and so forth, until they are no longer changing. $\theta_{2}^{\prime }$ and $\theta_{3}^{\prime }$ at this time are regarded as joint-angles under ideal mapping in the current position coordinate. According to the joint parameters *L*_2_ = 15 cm and *L*_3_ = 15 cm, the input/output curve surface of the forward/inverse kinematics model are shown in Figure 4. N sets of data are selected as the training sample from it. Due to the base-joint angle $\theta_{1}^{\prime }$ with correction as a precise value, only the values of $\theta_{2}^{\prime }$ and $\theta_{3}^{\prime }$ need to be solved, so two LS-SVM mapping models are established. Then, the desired trajectory position points are used as inputs of the trained LS-SVM mapping models. The outputs after calculation according to the models are the joint angles corresponding to the desired positions. The process of the inverse kinematics solution by LS-SVM is shown in Figure 5.

### 3.4. Results and Error Analysis 

**Figure 6:**
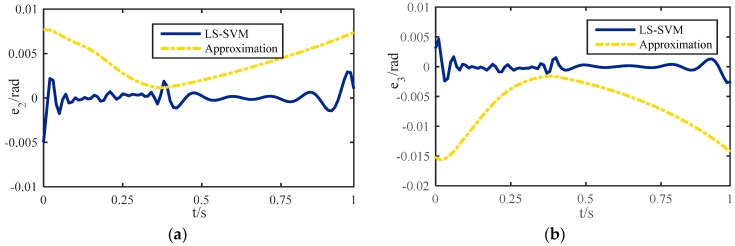
Joint angle errors of LS-LSM and the approximation method: (**a**) The angle error of the hip joint; (**b**) The angle error of the knee joint.

In our experiments, the step size is 0.10 m, the leg lift height is 0.05 m, the foot radius is 0.02 m and the land coefficient is 0.6 in one gait cycle. The number of training samples is N = 400, the input of the training samples are Cartesian coordinate (T^Ix0,T^Iz0) of each point in the desired trajectory, and the output of the training samples is $\left(\theta_{2}^{\prime },\theta_{3}^{\prime }\right)$ obtained by multi-iteration. The number of test samples is *n* = 200, the kernel function is a radial basis function, *γ* = 100, and *σ*^2^ = 0.2. The foot correction algorithm included trained model is used to solve the inverse kinematics of the robot with a semi-round rigid foot. Figure 6 shows that the maximum errors of $\theta_{2}^{\prime }$ and $\theta_{3}^{\prime }$ obtained by the approximate calculation are only 0.008 rad and 0.016 rad, but the errors can be reduced to 0.003 rad and 0.005 rad by using LS-SVM. Although the iteration algorithm can provide a better tracking accuracy, the execution cycle is 0.27 ms, while after the use of LS-SVM, this cycle is 0.15 ms. 

## 4. Experiments and Discussion

In order to verify the proposed trajectory correction methodology, a series of experiments were carried out on a leg platform and a hexapod walking robot. The experimental results and related discussion are presented in this section.

### 4.1. Single Leg Platform Tests

**Figure 7:**
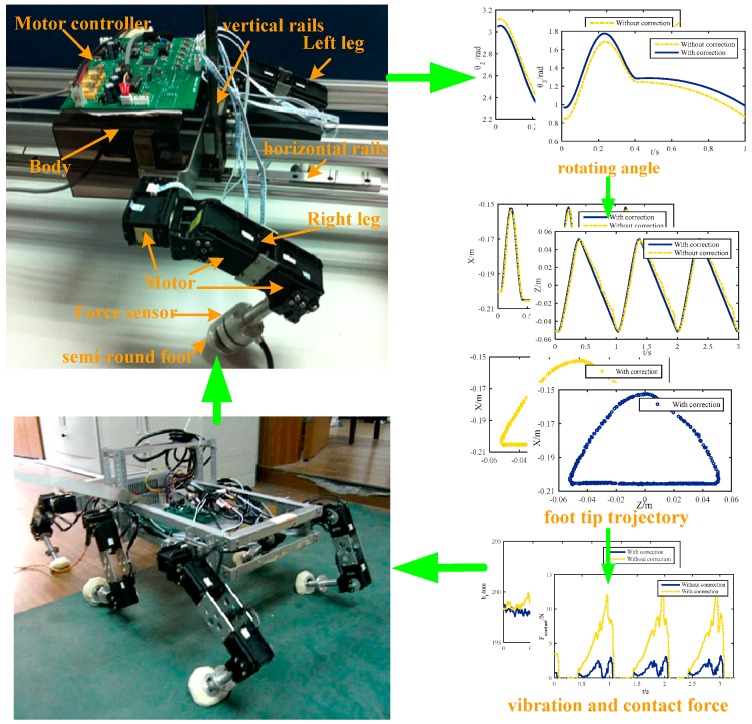
Leg platform test diagram for locomotion of the walking robot.

Experiments on a leg platform with less influence from the factors of the other legs are conducted and prove that the proposed methodology can not only correct the actual trajectory but also improve the leg locomotion. The platform is shown in Figure 7. 

The main body of the platform is supported with two legs in a mammal-like configuration, similar to the mentioned hexapod in Figure 1. Each leg consists of three linkages which are connected by a hip joint and a knee joint. The leg mechanism is attached to the body via a base joint. Since the platform is designed for locomotion tests, it is equipped with horizontal and vertical rails with corresponding displacement sensors, a five-axial force sensor, and LPC2368 based motor controllers. During the experiment of a single leg, the left leg is fixed on the supporting platform. Thus, the forward direction Z remains stationary, the X direction is free, and its displacement can be obtained by the vertical displacement sensor. The right leg moves in the desired trajectory periodically. The joint angle curves obtained by the proposed correction methodology and traditional method are used for the foot trajectory test, base-joint vibration test, and contact force test, as shown in the leg platform test diagram in Figure 7. According to the test results above, the performance of the methodology is observed. The gait parameters used in the experiments are as follows: the leg lift height is h = 0.05 m, the coefficient of land is β = 0.6, the step cycle is T = 1 s, the step size is S = 0.1 m, the body height is H = 0.2 m, and the radius of the semi-round rigid foot is R = 0.02 m.

**Figure 8:**
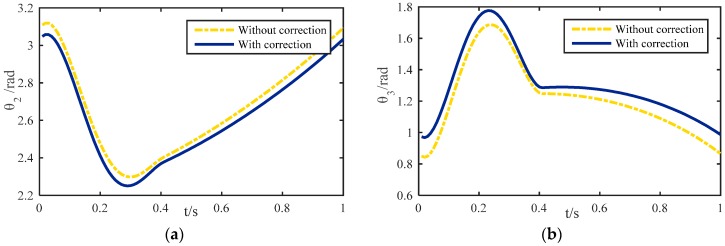
Rotating angle curves of different joints: (**a**) Rotating angle of the hip joint; (**b**) Rotating angle of the knee joint.

According to the forward/inverse kinematic methodology based on LS-SVM introduced above, if the gait of the robot is known, the corresponding joint-angle curves can be obtained. Finally, they can drive the robot to launch a series of experiments. Here, a tripod gait and an improved wave gait [37,38] are adopted to generate straight walking joint-angle curves. The corrected curves of each joint-angle and the uncorrected curves in a gait cycle are shown in Figure 8. Since the rotation of the base-joint is unchanged during straight walking, the angle curve of the base-joint is omitted. As for the tripod gait and the improved wave gait, if the parameters of those gaits are the same, the legs will have a similar motion under the two kinds of gait. Therefore, the joint-angle curves shown in Figure 8 are the same for the two gaits mentioned above.

#### 4.1.1. Foot Tip Trajectory

**Figure 9:**
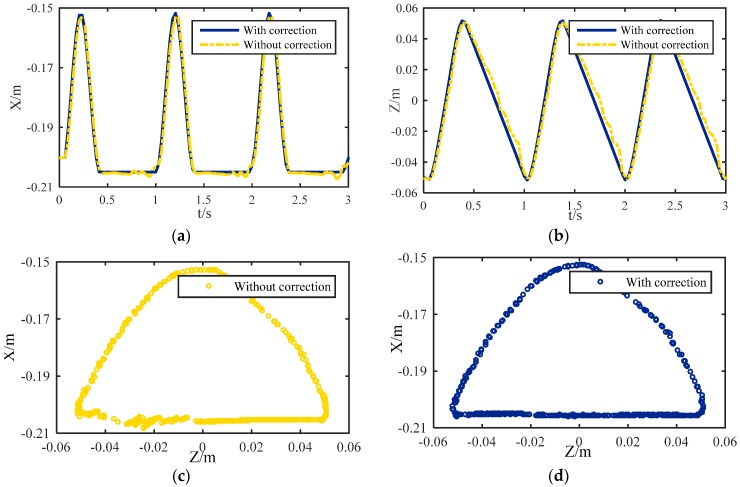
Trajectories of the foot tip relative to the base-joint: (**a**) Displacement in X-direction; (**b**) Displacement in Z-direction; (**c**) Synthesize Trajectory without correction; (**d**) Synthesize Trajectory with correction.

The trajectories of the foot tip relative to the base-joint are tested, which is shown in Figure 9. The supporting phase and the swing phase appear alternately and periodically. The gait cycle time is 1 s, and the leg is in swing state from 0 to 0.4 s. For the first half of that time period the leg is in lift-off phase, and in the second half it is in flight phase. The maximum lift height of the leg is 0.05 m as designed. From 0.4 s to 1 s, the robot is in the supporting phase. The displacement in the X direction is shown in Figure 9a. When the leg is in this state, the distance between the lowest point of the foot tip and the base-joint is larger than 0.2 m slightly. The trajectory in the X direction is not a smooth straight line, but has some fluctuation. That is because the actual contact point is not the design point. Thus, the robot will vibrate up and down during walking. However, with correction the trajectory becomes a smooth straight line. In the Z direction, the problem is the same as in the X direction, which is shown in Figure 9b. With correction, the smoothness of the trajectory is notably improved. The synthesized trajectory curves in Figure 9c,d are obtained by compounding the displacement of the X direction and the Z direction. The upper part of the curve represents the swing phase, and the lower part indicates the supporting phase. The unsmooth contact will make an impact on the ground and increase the contact force. Moreover, the imbalance body posture will cause slipping of other legs. With correction, the contact trajectory is a smooth straight line. The centroid of the robotic body will be kept constant during movement, so the robot will be more stable during walking.

#### 4.1.2. Base Joint Vibration

**Figure 10:**
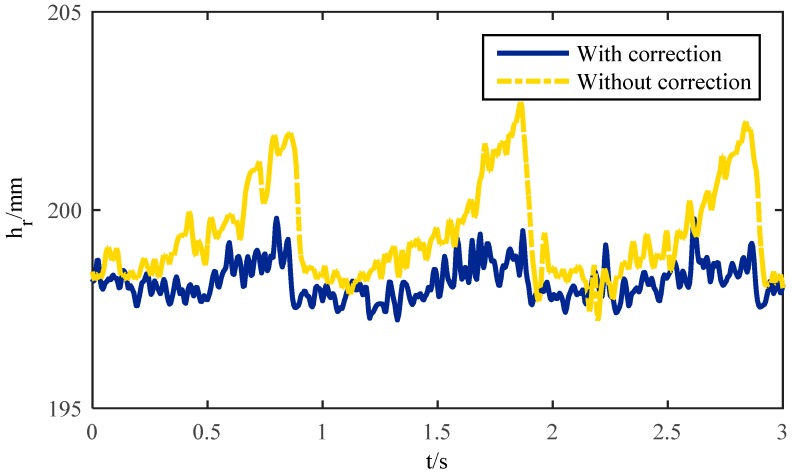
Base joint vibration of a single leg.

The base-joint is connected with the body rigidly, and the trajectory of the centroid of the robot is determined by the trajectory of the base-joint. The vibration of the base-joint will affect the motion of the robotic centroid directly. If the vibration is large, the energy consumption of the robot will be increased and the stability will be greatly reduced. According to the experiments above, the corrected trajectory of the base-joint in the X direction and the un-correction trajectory are tested, which are shown in Figure 10. The ideal base joint trajectory according to the method above should be a straight line, since the robot should go forward along the Z-axis with constant speed and no displacement in the X direction. It can be concluded from Figure 10 that the trajectory without correction has apparent vibration in the X direction and the value of the deviation is nearly 5 mm at the time points 0.9 s, 1.8 s, and 2.7 s. That means the robot will suffer periodic shocks while moving. However, after correction, the base-joint trajectory is almost kept level, the vibration is small, and the stability is improved. This result is not only related to the radius of the semi-round foot but also to the angle between the end-linkage and the ground generated by the foot trajectory planning. In the second half of the support phase, as the angle between the end-linkage and the ground increases, the distance of the actual contact point and the ideal contact point becomes farther. This makes the base-joint more shock upwards obviously. The trajectory correction methodology proposed can be used for effectively modifying the trajectory of the base-joint.

#### 4.1.3. Contact Force

**Figure 11:**
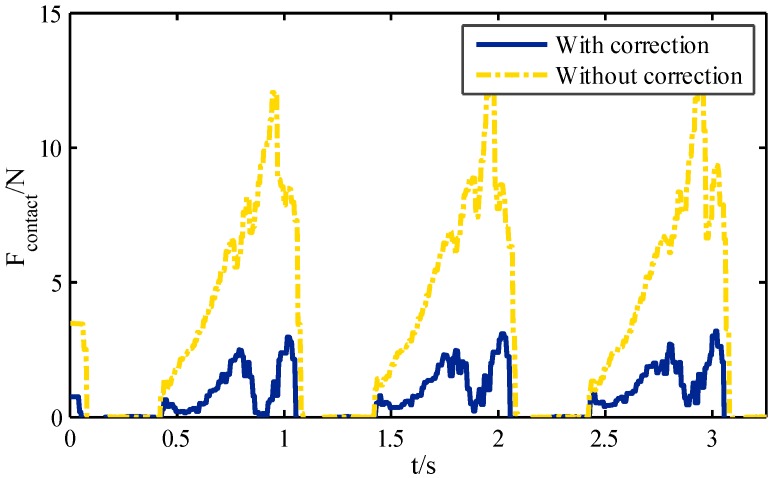
Contact force of a single leg.

During the movement of the hexapod robot, the foot mechanics play a very important role [39]. On unstructured terrain, the robot can adapt to complex conditions by changing its posture, and the gait generation strategy of the robot according to the foot tip contact force. The study of foot mechanics is beneficial to optimize the gait planning, enhance the stability, and reduce the energy consumption of the robot during locomotion [40]. In the single leg experiment shown in Figure 7, the ideal state is that in which the contact between the foot and the ground is kept in a critical state. However, if there is a deviation between the real trajectory and the desired trajectory, it will lead to interaction with the ground, causing a bigger contact force or even impact, which is the reason for vibrations. If the foot trajectory is designed in a better way, the force will be smaller and with no sudden changes, which demonstrates that the contact stability is enhanced. If there is no force, it means no contact and it is unreasonable as well. The forces with correction and without correction are all tested in experiments, which are shown in Figure 11. It illustrates that the foot starts touching the ground at 0.4 s and the contact force without correction becomes larger, which corresponds with the trajectory deviation. At time points 0.9 s, 1.8 s, and 2.7 s, the force reaches a maximum of 13 N, but with correction, the trajectory deviation becomes smaller. Meanwhile, the contact force is also very small, reduced by 70% compared with the uncorrected force, and less than 4 N, which can be considered as a critical contact with the ground. That means that the modification of the trajectory by the suggested methodology can reduce the influence on the contact force effectively. This has great significance for the further control of the foot force and stability [41]. 

### 4.2. Walking Tests of the Hexapod Robot

After the single leg experiments described above, walking experiments of the hexapod robot were carried out. The overall structure of the hexapod robot system is shown in Figure 1. The axis of the hip-joint is parallel to the forward direction of the robot. The robot consists of its body and six legs. Because the six legs are distributed symmetrically along the two sides of the robotic body, the location of the geometrical center can be regarded as the location of gravity of the robot. As mentioned before, tripod gait and improved wave gait were both adopted to generate joint angle curves in straight walking. The parameters of the following tests are the same as the leg platform tests.

**Figure 12:**
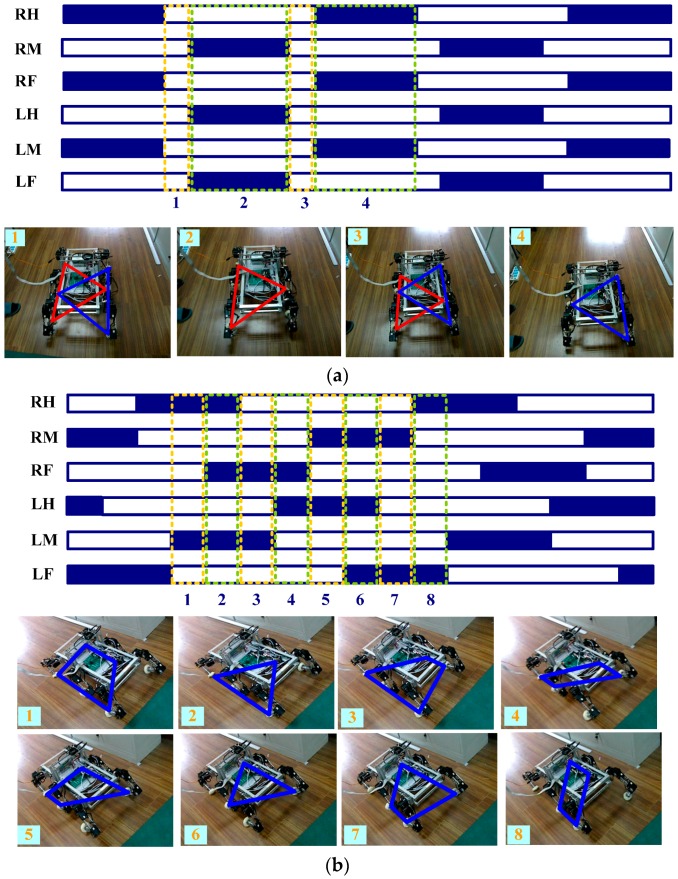
Diagram and snapshots of different gaits: (**a**) Tripod gait; (**b**) Improved wave gait.

The robot walking with a tripod gait is shown in Figure 12a. The blue section represents the swing phase, and the white section represents the supporting phase. In this gait, the legs are divided into two groups (LF, RM, and LH are the first group; RF, LM, and RH are the second group). Two groups alternately appear in supporting state and swing state. Because the landing coefficient is 0.6, the six legs will support at the same time in a moment of 0.2 s in 1 s. The initial state of the robot is shown in snapshot 1, where the six legs are all in the supporting state. In snapshot 2, the first group of legs (marked by red) is in the supporting state and second group is in the swing state. In snapshot 3, the six legs are all in the supporting state, prepared for gait alternation. In snapshot 4, the second group of legs (remarked by blue) is in the supporting state, and first group is in the swing state. At this point, one gait cycle is completed. The diagram of the gait and snapshots are respectively shown in Figure 12b when the robot is walking with improved wave gait. The mechanical legs located in the vertices of the blue polygon are in the supporting state. The rest of the mechanical legs are in the swing state (for instance, in snapshot 1, RH and LM are in the swing state, and RM, RF, LH, and LF are in the supporting state).

#### 4.2.1. Direction Deviation

**Figure 13:**
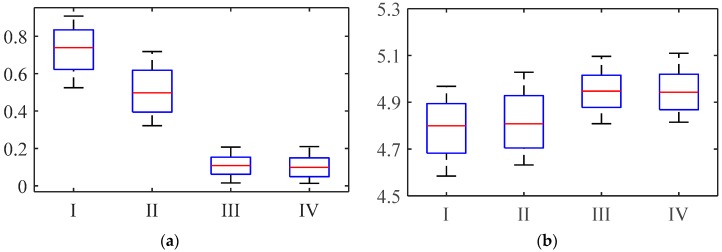

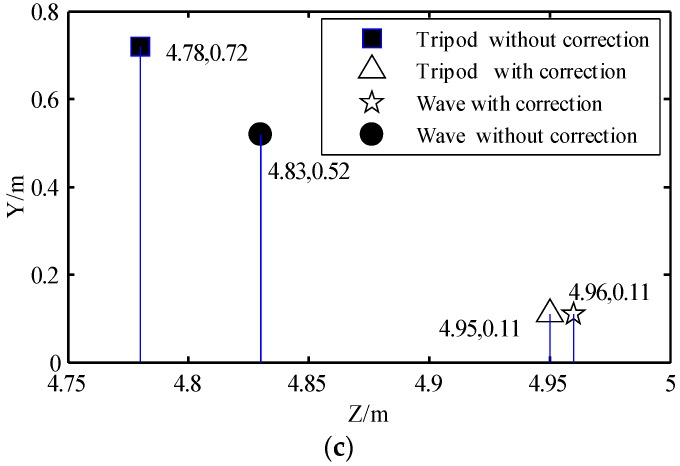
Walking experiment results of the hexapod robot: (**a**) Deviation in Y-direction; (**b**) Progress in Z-direction; (**c**) Position deviations. **I**, **II**, **III**, **IV**, respectively represent tripod gait without correction, improved wave gait without correction, tripod gait with correction, and wave gait with correction.

Because of the terrain or slippage of certain legs, the robot will appear to deviate from its direction during movement. In this part, the experiments are carried out on smooth ground, as shown in Figure 12. Thus, the influence of the ground can be excluded. In order to verify the effect of the correction methodology on the deviation, the deviation in the Y and Z directions with and without correction are compared. The deviations of the two gaits with the two methods in the Y-direction are shown in Figure 13a. In the diagram, for the tripod gait without correction, the minimum deviation distance is 0.52 m, the maximum is 0.92 m, and the mean value after 10 tests is 0.72 m. Similarly, for improved wave gait without correction, the values of deviation are slightly smaller, because it has more legs supporting it than with the tripod gait. However, for the gaits with correction, the minimum deviation distance is 0.02 m, the maximum deviation distance is 0.21 m, and the mean value is 0.11 m. The slippingage was very serious and obvious for some feet while walking, when the joint trajectory without correction was used. It caused the robot to deviate from the desired direction and trajectory. After using the correction methodology, the deviation distances of the tripod gait and improved wave gait are reduced on average by 84% and 78%, respectively. The progress in the Z-direction is shown in Figure 13b. The progresses of both gaits with correction are all slightly larger than before. Since the foot slippage is reduced, larger friction is obtained and the locomotion of the robot is dramatically improved. The position deviations under the two methods are shown in Figure 13c.

#### 4.2.2. Energy Consumption

The system energy consumption of the robot under two different methods during walking is studied based on the analysis above. The energy consumption of each leg in the robot system is evaluated by measuring the current of each leg during walking.

**Figure 14:**
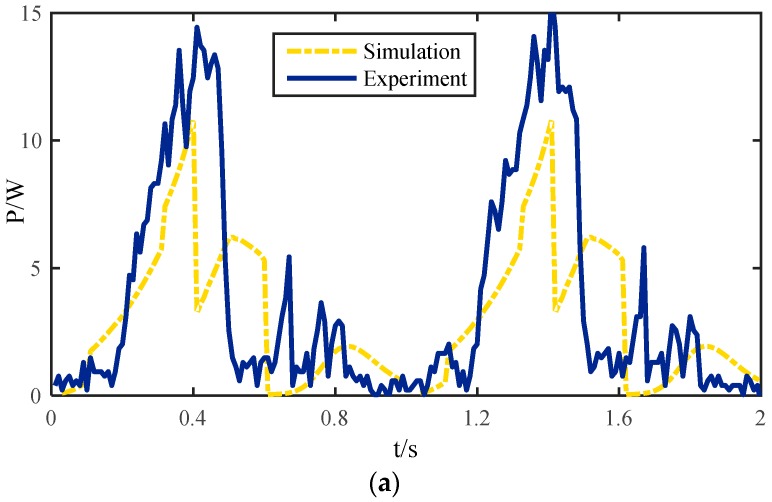

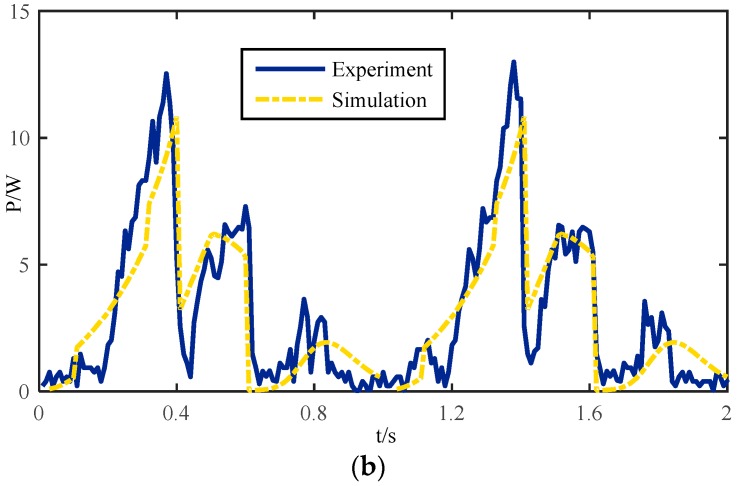
Energetic cost curves of a single leg: (**a**) Without considering the shape of foot; (**b**) Correction algorithm with a semi-round rigid foot.

The energetic cost curves of a single leg in improved wave gait with two methods are shown in Figure 14. The dashed line represents the optimized theoretical value. The solid line in Figure 14a represents the energy consumption of a single leg under control of the kinematic algorithm without taking foot shape into consideration. The solid line in Figure 14b represents the energy consumption by using a semi-round rigid foot correction methodology. As can be seen from the graph, without the use of the modified algorithm, the maximum value of the actual power exceeds 15 W, while the theoretical maximum value is 12 W. However, with the correction methodology, the actual maximum power is 13 W, which is very close to the theoretical value. The trend of the curve is more consistent with the theoretical value. That is because the slippage of the legs at some times will affect the trend of energy consumption. On the other hand, the slippage will also lead to redistribution of the contact force and moment of the leg, and eventually affect the energy consumption results of the whole system, so taking the foot shape into consideration during the trajectory planning has great significance for stability. 

**Figure 15:**
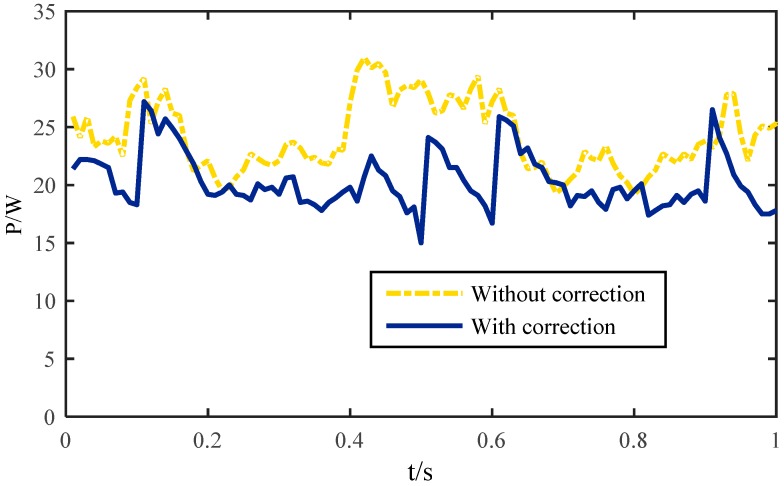
System energy consumption of the robot.

A comparison of system energy consumption between the correction methodology (dashed line) and un-correction (solid line) in one gait cycle time is shown in Figure 15. It is obvious that the maximum power is 31 W without correction and 27.5 W with correction. The average value of the energy consumption goes from 25 W to 20 W. That means about 20% of the energy can be saved during walking, so the correction methodology not only avoids the force impact caused by sliding of the supporting legs, but also reduces unnecessary energy consumption caused by interference of each leg with the semi-round foot.

## 5. Conclusions

In this paper, the body trajectory misplacement problem caused by the rolling of a robot foot with a semi-round structure is analyzed. The influences of joint trajectory generation, foot tip trajectory, base joint vibration, contact force, body trajectory and energy consumption caused by the semi-round structure during robot movement are studied. Through the analysis of the forward/inverse kinematics of the robot leg, a correction methodology for a robot with a semi-round rigid foot based on LS-SVM is proposed. According to the results, the suggested method has higher accuracy than the approximation method used before, and has a faster calculation speed of the inverse kinematic solution than the iterative method. The locomotion analysis of the robot obtained by comparing correction methodology under a semi-round foot based on LS-SVM with a traditional generation indicates that the correction methodology can effectively improve the foot tip trajectory, so as to reduce the influence of the semi-round foot on the vibration of the base-joint and the impact of contact force. The validity of the correction methodology for the semi-round rigid foot is verified by conducting experiments on a hexapod walking robot. Using tripod gait and improved wave gait as examples, the results indicate that the correction methodology can reduce slipping and trajectory deviation of the robot. The current detection also demonstrates that the proposed methodology can reduce the system energy consumption to some extent. Therefore, the correction methodology has great significance for the locomotion performance and the stability of walking robots.

The theoretical contributions and novelty of this paper can be summarized as follows. An approach for the misplacement problem caused by rolling of a foot with a semi-round structure is proposed. A solution method based on LS-SVM with higher accuracy and faster calculation speed is also established. Influences of joint trajectory generation, foot tip trajectory, base joint vibration, and contact force caused by the semi-round structure are studied. Finally, a series of experiments are performed on a hexapod robot, and the locomotion of the robot is studied using the proposed method. Slipping, trajectory deviations, and system energy consumption of the robot are all reduced, which validates our theory. In addition, the suggested methodology can be used as an underlying control of the robot architecture and the theory is concise and straightforward in the software, which indicates that force control, gait generation, trajectory planning, and other advanced theories can be applied simultaneously. This means that further improvement can be performed without too much effort.

## Figures and Tables

**Table 1:** Denavit-Hartenberg parameters.

Link *j*	*α_i__(__j__-1)_*/°	*a_i__(__j__-1)_*/m	*θ_ij_*/°	*d_ij_*/m
1	0	0	−30°~60° (*i* = 1, 2, 3)/−60°~30° (*i* = 4, 5, 6)	0
2	90°	0	−240°~0° (*i* = 2, 3, 4, 5)/0°~240° (*i* = 1, 6)	0
3	0	*L*_2_	−120°~0° (*i* = 2, 3, 4, 5)/0°~120° (*i* = 1, 6)	0
